# Physical losses could partially explain modest carotenoid retention in dried food products from biofortified cassava

**DOI:** 10.1371/journal.pone.0194402

**Published:** 2018-03-21

**Authors:** Aurélie Bechoff, Keith Ian Tomlins, Ugo Chijioke, Paul Ilona, Andrew Westby, Erick Boy

**Affiliations:** 1 Natural Resources Institute, University of Greenwich, Chatham Maritime, Kent, United Kingdom; 2 National Root Crop Research Institute, Umudike, Umuahia, Abia State, Nigeria; 3 HarvestPlus Nigeria, c/o International Institute of Tropical Agriculture (IITA), Ibadan, Oyo State, Nigeria; 4 HarvestPlus Headquarters, c/o IFPRI, NW, Washington, DC, United States of America; Agriculture and Agri-Food Canada, CANADA

## Abstract

Gari, a fermented and dried semolina made from cassava, is one of the most common foods in West Africa. Recently introduced biofortified yellow cassava containing provitamin A carotenoids could help tackle vitamin A deficiency prevalent in those areas. However there are concerns because of the low retention of carotenoids during gari processing compared to other processes (*e*.*g*. boiling). The aim of the study was to assess the levels of true retention in *trans*–β-carotene during gari processing and investigate the causes of low retention. Influence of processing step, processor (3 commercial processors) and variety (TMS 01/1371; 01/1368 and 01/1412) were assessed. It was shown that low true retention (46% on average) during gari processing may be explained by not only chemical losses (*i*.*e*. due to roasting temperature) but also by physical losses (*i*.*e*. due to leaching of carotenoids in discarded liquids): true retention in the liquid lost from grating negatively correlated with true retention retained in the mash (R = -0.914). Moreover, true retention followed the same pattern as lost water at the different processing steps (*i*.*e*. for the commercial processors). Variety had a significant influence on true retention, carotenoid content, and *trans*-*cis* isomerisation but the processor type had little effect. It is the first time that the importance of physical carotenoid losses was demonstrated during processing of biofortified crops.

## Introduction

An insufficiency of vitamin A in the diet results in vitamin A deficiency (VAD). VAD is responsible for night blindness, increased susceptibility to infections, impaired growth and development and remains a major public health issue in many developing countries, with children and pregnant/lactating women being the most vulnerable [1]. Cassava is a major root crop in Low and Middle Income Countries [2]. In Nigeria, the most densely populated country in Africa and the world largest cassava (*Manihot esculenta Crantz*) producer, the prevalence of low serum retinol among children 0–59 months of age is 30% [1]. The consumption of cassava is high, being approximately 600 grams per person per day (fresh weight) on average [3]. Hence the introduction of biofortified cassava varieties with yellow coloured roots that contain significant amounts of provitamin A carotenoids (pVACs) gives strong hope that these biofortified cassava varieties could tackle VAD in West Africa and other developing countries [4, 5].

Gari, a fermented granulated food—that may have prebiotic [6] or probiotic [7–9] beneficial activity—is the most popular food product made from cassava in Nigeria and West Africa and its production represents two thirds of the cassava grown [3, 10]. When made from biofortified cassava, gari has a distinct yellow colour and is visually similar to a type of local gari made with added palm oil that is well accepted in some parts of Nigeria [11, 12].

Measuring the retention of provitamin A during processing is critical in order to ensure that the biofortified food retains sufficient pVACs and hence has health benefits for the people who will consume it. The determination of True retention (TR) is important because it takes into account the changes in the weight of food during cooking (for example, water loss; losses of soluble solids) and gives a fairer estimate of the actual carotenoid retention during the process. However, TR is more complex to determine than simple carotenoid content because it requires the weight of the product (*e*.*g*. cassava made into gari) to be followed throughout processing.

Processing cassava into foods such as gari usually involves several processing steps due to the need to remove the cyanide content inherent to the root [10], reduce the water content, and ferment in order to develop the desired product characteristics. A challenge faced with such lengthy processes and with biofortified crops such as yellow cassava is that pVACs are chemically unstable molecules that can be degraded during processing and storage. Chemical loss occurs through two different mechanisms: 1) *trans*-*cis* isomerisation and 2) oxidation. Chemical degradation is typically caused by temperature, oxygen and light exposure [13]. The mechanisms of temperature and oxygen degradation were described in the case of storage of dried orange fleshed sweet potato [14, 15]. As well as being chemically degraded, pVACs can be physically lost during processing (*i*.*e*. in moisture removed from the product) but less is known about the extent of these losses and their impact.

Higher reduction of pVACs from biofortified yellow cassava during gari production compared to most other processes such as boiling, oven drying, and frying has been demonstrated by several authors [16–23]. However studies on gari retention were conducted under experimental conditions either in a laboratory or in a relatively small scale processing, or with insufficient levels of details at processing steps. In a research work on gari in Nigeria [20], changes in total carotenoid content were reported at different stages of gari processing on an on-station processing plant with small quantities of roots (10kg) and no processing replicates; the levels of true retention (TR) were not reported. In another study [21], working on a similar scale and setting than the previous study [20], TR of total carotenoids in the final product (gari) was 45% on average for three cassava varieties processed in triplicate, but the TR levels at the different processing steps were not indicated. Thakkar et al. [22] determined the different carotenoids present and their concentration in a laboratory-scale experiment. Although the authors indicated that TR was 51% on average for three yellow-fleshed varieties, TR levels were not broken down for the different processing steps. Chavez et al. [16] also studied carotenoid retention during gari production in the laboratory and reported that TR of *trans*-β-carotene was 34% for three cultivars with three replications. However gari was fermented for 7 days which is longer than fermentation times in West Africa (typically 2–3 days). *Trans*-β-carotene contents and retention were determined by Failla et al. [18] in a study on the retention of β-carotene in transgenic roots of yellow cassava. Conversely La Frano et al. [19] worked with a conventionally bred cassava variety from Nigeria (07/0593). Retention was approximately 40% in these studies under laboratory conditions but it is not known if the calculation of retention was based on the fresh weight of the sample and was indeed true retention (TR). In addition, in those studies [18–22], carotenoid losses were generally attributed to chemical factors such as isomerisation and oxidation and physical losses were not clearly mentioned.

It appears that there are gaps in knowledge on the levels of TR during processing of cassava into gari: previous research on the level of true retention (TR) of pVACs during gari processing has been mainly under set conditions and/or only on global TR therefore limiting the understanding of the factors responsible for carotenoid loss. In addition there has been little investigation on the importance of physical losses of carotenoids. What is now required is a study to understand better the factors responsible for carotenoid loss that include an investigation of physical losses. This knowledge could ultimately lead to a reduction of provitamin A carotenoid losses during processing of gari.

In order to best understand conditions occurring in a field situation, our approach was to record the actual processing conditions rather than fixing these conditions; and measure the impact of field conditions on carotenoid retention. This is the first time that such an approach has been reported on carotenoid retention during gari processing.

Using different processors and varieties is important because processing conditions vary from one processor to another and varieties also might give different responses. Additionally we measured the carotenoid content and *trans*-*cis* isomerisation during processing in order to give a more complete picture of the changes in carotenoid during gari processing.

## Materials and methods

### Cassava root supply for experiments A and B

Roots of biofortified yellow varieties of the first wave (TMS 01/1371; 01/1368; and 01/1412) developed by IITA in collaboration with HarvestPlus were used in this study. No specific permissions were required because HarvestPlus/IITA had the authorisation to use those lands for research purposes. The study did not involve endangered or protected species.

There were two types of experiments: an experiment with commercial gari processors (Experiment A) and a varietal trial conducted with three different varieties over two seasons and locations (Experiment B) (Table 1).

**Table 1:** Parameters recorded during gari processing for Experiments A^a^ and B^b^.

Experiment		A			B (SL1)			B (SL2)		
Variety		01/1371	01/1371	01/1371	01/1368	01/1371	01/1412	01/1368	01/1371	01/1412
Place		Atiba	Barracks	Iseyin		IITA			IITA	
**pH after fermentation**		4.2±0.0bc	4.9±0.0d	4.1±0.0ab	4.0±0.0ab	4.4±0.1c	3.9±0.1ab	4.0±0.0ab	3.9±0.0ab	3.8±0.1a
**Temperature after fermentation (ºC)**		25.2±0.9bc	25.0±0.8bc	26.1±1.1d	25.7±0.6cd	25.7±1.2cd	27.7±1.2a	22.8±0.0ab	23.0±0.3ab	22.4±0.9a
**Time (h)**	**Peeling**	0.28±0.05ab	0.79±0.19c	0.31±0.03ab	0.30±0.02ab	0.20±0.01a	0.27±0.03a	0.65±0.03bc	0.54±0.16abc	0.44±0.21abc
	**Washing**	No	0.09±0.02a	0.09±0.02a	0.06±0.01a	0.06±0.01a	0.06±0.02a	0.06±0.02a	0.07±0.03a	0.04±0.01a
	**Grating**	0.04±0.02a	0.05±0.00ab	0.03±0.01a	0.12±0.01c	0.11±0.01c	0.09±0.02bc	0.03±0.00a	0.03±0.01a	0.03±0.01a
	**Fermenting**	46.62±0.15e	3.11±0.60a	66.58±0.12f	43.12±0.20cd	42.59±0.13bc	43.86±0.34cd	42.3±0.02bc	41.94±0.12b	42.79±0.12c
	**Pressing**	1.19±0.10a	1.50±0.00ab	1.38±0.00ab	1.68±0.35bc	1.92±0.00c	1.20±0.00a	3.50±0.00d	3.50±0.00d	3.50±0.00d
	**Sifting**	0.02±0.00a	0.24±0.03c	0.02±0.00a	0.04±0.01ab	0.05±0.00ab	0.06±0.02b	0.02±0.00a	0.02±0.00a	0.02±0.00a
	**Roasting**	0.43±0.05b	1.42±0.08d	0.68±0.04bc	0.23±0.01a	0.23±0.01a	0.22±0.01a	0.59±0.10bc	0.78±0.18c	0.57±0.09bc
	**Sieving**	0.07±0.02a	0.04±0.01a	0.08±0.08a	0.03±0.00a	0.03±0.00a	0.03±0.00a	0.12±0.06a	0.09±0.01a	0.08±0.04a
**Equipment**	**Grater**	Diesel-powered rotating grating machine—locally fabricated	Electricity or diesel-powered rotating grating machine	Diesel-powered rotating grating machine—locally fabricated	Diesel-powered rotating grating machine, Dandrea Agriport Industrias Maquinas d'Andrea (Brazil)					
	**Press**	Hydraulic jack type	Hydraulic jack type	Screw jack manual type locally made	32t -hydraulic jack type with wooden platforms					
	**Roaster**	Rectangular pan made from iron	Two round pans made from iron	Rectangular pan made from iron	Rectangular pan made from stainless steel iron with chimney					

Data are average ± standard deviation. Each process was conducted in triplicate

^a^ Triplicate 50kg of roots of one variety of yellow cassava TMS 01/1371 were processed into gari at three commercial gari processors (Atiba, Barracks and Iseyin) (Experiment A) and

^b^Triplicate 25kg of roots of three different varieties of yellow cassava (01/1368; 01/1371; 01/1412) grown in two different seasons/locations (S1 and S2) were processed into gari at the IITA research station (Experiment B). Fermented mash was not collected at the Barracks. Different letters in raws are significantly different data at p<0.05 (Tukey test; One-Way ANOVA).

In Experiment A, only one variety of biofortified cassava (TMS 01/1371) was used. The initial raw material was the same for all of the commercial processors. The root supply (500kg of roots) was from a field belonging to HarvestPlus at Ikenne (6◦86N, 3◦71E) [24]. TMS 01/1371 roots were harvested approximately 12 months after planting.

In the varietal trial (Experiment B), three varieties of biofortified cassava (TMS 01/1214; TMS 01/1368 and TMS 01/1371) were grown at two different seasons on separate locations. Having different locations and different seasons was useful to appreciate concomitant variation in the field and across seasons. The three varieties for the first season (SL1) (warm season) were grown on a field owned by IITA/HarvestPlus at the IITA research station in Ibadan (7◦38N, 3◦89E) [24]. These three varieties (about 100kg per variety) were harvested approximately 12 months after planting in September 2012. In the second season (SL2) (cold season), the three varieties were planted and harvested (about 100kg per variety) from Liji Farms, Ilero (8°40N, 3°21E) in July 2013. For logistical reasons Experiment B was conducted on a processing plant located in a research station. However the processing conditions and equipment were not very different to those used in Experiment A. In Experiments A and B, processing conditions were recorded the same way, by observation of local processors’practices.

### Processing of roots

Roots were processed on the day after the harvest. Each manufacture was carried out in triplicate.

In Experiment A, harvested roots from one variety (01/1371) were divided into the three different commercial processors (50kg processed in triplicate per processor) located in Oyo State, Nigeria. These were a) Atiba in Oyo (about 1h drive north from the International Institute for Tropical Agriculture (IITA)); b) Army Barracks in Ibadan, Ogo Oluwa Centre (less than 0.5h drive from IITA), and c) Crown Centre, Iseyin (about 1.5h drive north from IITA). These processors were selected by the Agricultural Development Program in Nigeria on the basis of having distinctive practices that were representative of the variability of processes existing in Oyo state.

Processing of roots for the three processors was initiated on the same day and under the same conditions of ambient temperature/humidity (27°C/70% on average).

In Experiment B, roots from three varieties (01/1371; 01/1368; 01/1412) were processed at the IITA processing unit (25kg in triplicate per variety). Roots for the three varieties were processed at the same time and therefore under the same weather (temperature/humidity) conditions.

The processing stages were the same for Experiments A and B: roots were peeled manually and washed with clean water to remove soil and particles. The peeled roots were then mechanically grated using a petrol engine-driven grater, packed into a polypropylene bag and left to ferment at ambient temperature. At the end of fermentation, mash in a woven bag that allowed water to drain was pressed using a hydraulic or manual press. The pressed mash was disintegrated (using the petrol engine-driven grater) in order to separate agglomerated particles. The sifted mash was then toasted in a steel pan heated by fire wood. Roasted granules that had been cooled down at ambient temperature for a few minutes were then manually sieved (4–5mm aperture sieve). Processing conditions were monitored in the field situation: a step-by-step observation and recording of the quantities, ambient temperature/humidity, length of time, pH values and temperature of the mash before and after fermentation and roasting temperature were carried out.

### Observation of the traditional processing practices

There were variations in the equipment and in practices; in particular between the commercial processors (Atiba, Barracks, Iseyin) (Experiment A) (Table 1). Atiba processors did not wash roots prior to peeling contrary to the other two processors. Fermentation time was significantly different for the three commercial processors and this significantly influenced pH value: the time of fermentation was the shortest at the Barracks (3h; pH = 4.9); 2 days at Atiba (47h; pH = 4.2) and 3 days (66h; pH = 4.1) at Iseyin. A manual press was used by Atiba and Barrack processors whilst those in Iseyin used a screw jack type- manual press. Sifting was done using a mechanised grater in Atiba and Iseyin whilst at the Barracks sifting was done by hand using a 4–5mm aperture-sieve. Atiba and Iseyin processors used non-stainless plates for roasting whilst at the Barracks, sifted mash was roasted in round shaped pans. Roasting time varied between 0.22and 1.42h.

In Experiment B, variations were minimal between the three varieties (these were processed by the same team), and this means that the varietal effect can be measured independently. There were however a few differences between processing in SL1 and SL2: in SL2 peeling, pressing and roasting times were significantly longer. Differences may be explained by difference in operators (*e*.*g*. peeling ability), root moisture content, and season: in particular, the average temperature of the mash after fermentation was lower in the cold season (SL2; 23°C) compared to the warm season (SL1; 26°C), and this may explain why pressing and roasting would have taken more time in the cold season.

### Analytical measurements

Samples were weighed during processing using a digital scale (EHF-203 Series Digital Hanging Scales, Scales of the World, Milton Keynes, UK) with a maximal load of 50.0 kg. In addition, the whole quantity of liquid lost from grating (‘liquid from grated mash’ or also locally known as ‘grated juice’) was collected in a basin separately to the mash and the quantity of liquid was weighed immediately after the grating process (to limit risks of evaporation and hence change in liquid quantity). The pH value was measured after fermentation using Hannah waterproof pH meter with dual LCD (Hannah Instruments, Leighton Buzzard, UK). Samples (10.0g) were weighed into a clean and dry container using an electronic balance (CS5000, Ohaus, I Parsippany, NJ, USA–maximal weight 5kg. readability 1g). Double the amount (= 20.0g) of distilled water was added and the sample stirred. The electrode of the pH meter was cleaned before pH value was recorded in the sample. An infra-red thermometer (RayTemp® 3, ETI, Worthing, UK) was used to measure product temperature. Time was recorded using the digital time on the mobile phone. Ambient temperature and humidity were recorded throughout processing using Tinytalk Ultra 2 device (RS Components Ltd, Northants, UK).

### Sample collection

Representative samples (100–150g) (peeled roots; grated mash; liquid fromgrated mash; fermented mash; fermented and pressed mash and sieved gari) were collected for moisture and carotenoid content determination. The peeled roots were collected as follows: three average-size roots were collected, peeled, quartered, chopped and mixed according to the method by Rodriguez-Amaya & Kimura [13].

### Sample storage and transport

Precautions were taken to keep samples as cool as possible and protect them from direct light exposure during collection and transport. Immediately after collection in the field, samples from each stage in the process were stored in good quality zip bags (heavy duty zipper LPDE 152 x 330) in a thermo insulated cool box packed with frozen gel. Samples of the liquid from grated mash were collected in 50ml polypropylene sample tubes hermetically closed with a screw top. Three liquid samples in SL2 were missing for collection. On return from the field each day, samples were placed in the freezer (-20°C).(aside freeze-drying, freezing is the best way of preserving carotenoids for analysis. The texture of the product can be changed by freezing but the total water content will be preserved). Samples were maintained frozen during air freight to the UK and stored in the freezer (-20°C) immediately upon arrival. Prior to carotenoid analysis, samples were allowed to thaw overnight in the refrigerator (8°C).

### Carotenoid analysis

The extraction stage was adapted from a previous method [25]. Analyses were carried out at NRI, UK. Dried samples (100–150g) (i.e. pressed mash and gari) were rehydrated for 10 min. in 10 ml deionised water. Fresh samples (i.e. peeled and chopped roots) were homogenised into a puree using a mechanical food blender (Kenwood type) and extracted without rehydration. In brief, a portion of the homogeneous representative sample (0.6–3.0g depending on the concentration of carotenoid and moisture in the sample) was homogenised with 50mL methanol:tetrahydrofuran (THF) (1:1) for 1 minute and filtered. The homogenised extract was rinsed with methanol:THF (1:1) until there was no yellow colour left in the filtrate. Partition between the aqueous phase and organic phase containing the carotenoids was achieved by addition of petroleum ether (PE 40–60° C) and NaCl solution (10%). The PE phase was further washed with deionised water, dried by addition of anhydrous sodium sulphate, then filtered and made up to volume (25 ml). Extracts were then dried by flushing with nitrogen in a dry block system at 35° C. Dried extracts were dissolved in 500 μl THF: Methanol (1:1). After vortexing, dissolved extracts were collected into a vial with septum for HPLC analysis. A reverse-phase high performance liquid chromatography using an Agilent 1200 system (UK) was used with a polymeric C30 reverse phase column (250 x 4.6 mm i.d. 5μm YMC (EUROP GmbH, Dinslaken, Germany) having a flow rate of 1 ml.min^-1^ a temperature of 25°C, a running time of 40 minutes and an injection volume of 10μl. The isocratic mix consisted of Methanol: MTBE (80:20). Detection of compounds was performed at 450nm. Concentrations on a fresh weight basis were determined by comparison to a standard curve using pure *trans*-β-carotene (Sigma, Dorset, UK). Percentages of *cis*-isomers and other minor compounds were also determined [26]. Molecular mass of *trans*-β-carotene (C40H56 = 536.87 g.mol-1) is identical to that of 9-*cis* and 13-*cis* of the same chemical formula (C40H56). Using a standard made with *trans*-β-carotene may therefore not make a difference in terms of the concentration of *cis*-isomers.

### True retention (TR)

True retention of *trans*-β-carotene (TR) was calculated according to Rodriguez-Amaya & Kimura [13]:

$$TR(\%)=100x\frac{trans-\beta -carotene\;content\;per\;kg\;of\;processed\;sample\;x\;weight\;of\;processed\;sample\;(kg)}{trans-\beta -carotene\;content\;per\;kg\;of\;peeled\;roots\;x\;weight\;of\;peeled\;roots\;(kg)}$$

*Trans*-β-carotene loss is: 1−*TR*(%)

True retention (TR) was calculated at the different steps of processing. The value in processed sample is expressed relative to the value of *trans*-β-carotene before processing (peeled roots). TR is based on the initial carotenoid quantity of the peeled roots (100%).

### Dry matter determination

Samples were collected and analysed for dry matter determination, at the same time as for carotenoid analysis. Determinations were made by drying triplicate 5 g samples at 105°C to constant weight (minimum 24h) [27]. Moisture content (%) is defined as: 1- dry matter content.

### Product yield (PY)

Product yield (PY) remaining at each step of processing was calculated by weighing the samples at the different steps of processing and dividing by the initial weight of unpeeled roots (50kg or 25kg).

$$\mathrm{PY}(\%)=100\;x\frac{weight\;of\;sample\;during\;processing\;(kg)}{initial\;sample\;weight\;(kg)}$$

Product yield (PY) is the percentage mass of the product that remains after each step and based on the initial mass of unpeeled roots (100%).

### Statistical analysis

Data were processed on SPSS 23.0 software for Windows using analysis of variance (ANOVA) and correlation test. Significant differences between data were assessed by a Tukey HSD test (p < 0.05). Significance of correlations was tested using a two-tailed Pearson test (p < 0.05).

## Results and discussion

### True retention during gari processing

#### Experiment A

**Fig 1:**
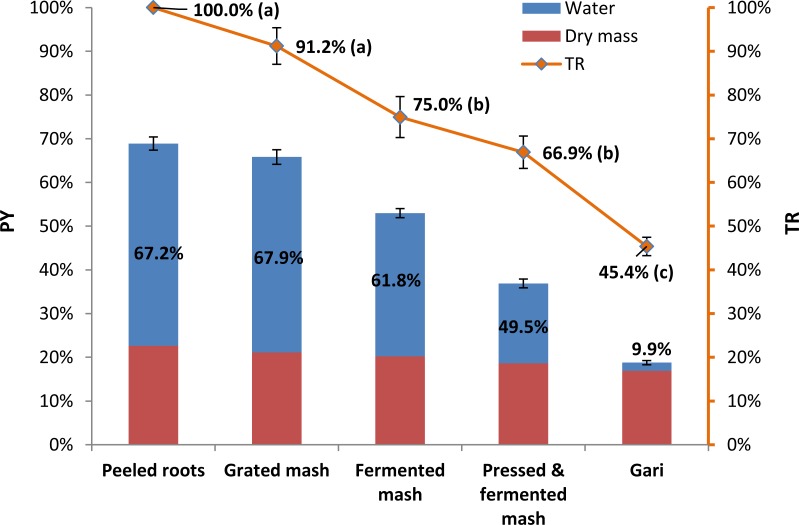
Schematic representation^a^ of true retention of trans-β-carotene (TR) during gari processing—Experiment A. ^a^Average and standard error (error bar) for 1 yellow cassava variety TMS 01/1371 at 3 commercial processors. Data for the three locations being Atiba, Barracks, Iseyin (Oyo State, Nigeria) are in triplicate for each location (n = 9). TR are represented in relation to the product yield (PY), dry mass and moisture. Different letters (a, b, c) indicate significant differences in TR between the steps of processing (ANOVA, Tukey test; p < 0.05). Product moisture content (%) is indicated in the blue area. The red area represents the dry mass of the product during processing.

Product Yield (PY) and True Retention (TR) during gari processing of the TMS 01/1371 variety at three commercial gari processors (Experiment A) are presented in Fig 1.

The cassava product is schematically represented as being partially composed of dry mass (dry part of the product) (DM) and of water or moisture.

There was no significant difference between TR in the three commercial processors (One-way ANOVA; p < 0.05). Hence each data point presented in Fig 1 is of the three processors combined and in triplicate (n = 9). The lack of overall difference in TR between the processors in spite of the different processing durations is an interesting finding because it shows that variation in processing parameters might not be preponderant for the degradation in carotenoids. In particular variation in fermentation length at the three commercial processors (3h, 47h, and 66h) did not significantly impact carotenoid degradation and this was in accordance with Thakkar et al. [22] and also with Onadipe Olapeju [28] who worked with the same cassava varieties in Nigeria.

On the other hand, there was a significant influence of the processing steps on TR (ANOVA, Tukey test; p < 0.05). Degradation of *trans*-β-carotene during gari processing followed a gradual loss with main losses (1- TR) occurring at fermentation and roasting. TR was not significantly different between peeled roots and grated mash (100%, and 91.2%, respectively), fermented mash and pressed mash (75.0% and 66.9% respectively), and gari had significantly lower TR (45.4%) than the other products.

TR at the final step, in gari (45.4% on average) was in accordance with previous retention studies on gari [16, 18, 19, 21, 22]; this would confirm that retention at commercial processors is similar to that found at smaller scales or laboratory conditions. Fig 1 clearly shows that gari processing is essentially a water removal process: during processing of cassava into gari, dry mass only slightly decreased (from 22.6% to 16.9%), whilst the moisture content was greatly reduced (from 67.2% to 9.9%) as well as PY (from 68.9% to 18.8%).

#### Experiment B

**Fig 2:**
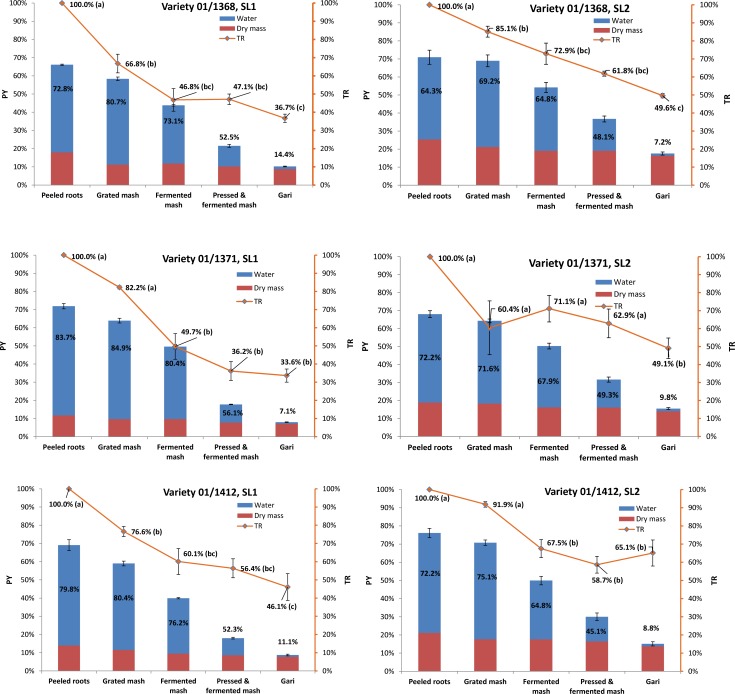
Schematic representation^a^ of true retention of trans-β-carotene (TR) during gari processing—Experiment B. ^a^Average and standard error (error bar) are for 3 yellow cassava varieties TMS 01/1368; 01/1371; 01/1412 processed in triplicate (n = 3) at 2 different seasons/locations (SL1 and SL2). TR are represented in relation to the product yield (PY), dry mass and moisture. Different letters (a, b, c) indicate significant differences in TR between the steps of processing (ANOVA, Tukey test; p < 0.05). Product moisture content (%) is indicated in the blue area. The red area represents the dry mass of the product during processing.

The influence of variety and season/location (SL) were explored (Experiment B). Variety and season/location (SL) both had significant influence on TR (ANOVA, Tukey test; p < 0.05) therefore the data were presented in separate graphs for the three varieties (01/1371; 01/1368, and 01/1412) and the two seasons/locations in years 1 and 2 (SL1 and SL2) (Fig 2).

It should be noted that in this experiment we were not able to separate out the effects of season and location because both varied from year 1 to year 2 but the additional variability is more representative of the field situation for gari processing as processors will experience concomitant seasonal and location variations.

On average, TR in gari was lower in SL1 than in SL2 (38.8%, and 54.6% on average, respectively). Hence there was an important influence of the season/location. The difference in TR between SL1 and SL2 might be explained by the difference in root moisture content that was higher in SL1 than in SL2 (78.8% and 69.6% on average, respectively). As a consequence, yield was much lower in SL1 than in SL2 (PY = 9.0% and 16.1% on average, respectively) (Fig 2). Amoah et al. [29] reported gari yields varying between 16 and 28% for gari from white cassava but yields for yellow cassava are known to be lower, as this was observed, in particular in SL1. Some authors have observed a linear relationship coexisting between loss in β-carotene during processing and initial dry matter content in roots: when investigating dried orange-fleshed sweet potato, Bechoff et al. [30] reported that moister roots (with a higher initial moisture content) had lower TR after drying. Ceballos et al. [31] similarly showed that TR in boiled cassava was negatively correlated to moisture content in the roots and this is in accordance with our results. We explain it because gari processing is essentially a process where moisture is removed and therefore this affects the weight of the product and hence there is a correlation between TR, PY and moisture content.

Variety also had a significant effect on TR (ANOVA, Tukey test; p < 0.05): final TR (in gari) for TMS 01/1371 variety (33.6% (SL1); 49.1% (SL2) being 41.4% on average) was not significantly different from that of 01/1368 variety (36.7% (SL1); 49.6% (SL2) being 43.2% on average) but significantly lower from that of 01/1214 variety (46.1% (SL1); 65.1% (SL2) being 55.6% on average). Maziya-Dixon et al. [21] working on three varieties of yellow cassava made into gari similarly reported varietal differences with TR for total carotenoids of 38.1; 49.8; and 46.8% for TMS 01/1371; 01/1235 and 94/0006 varieties, respectively. However those losses were not directly related to differences in dry matter content as in our present study. Further work is needed to understand the respective influence of variety and initial root dry matter content on TR in gari.

In addition to varietal and season/location (SL) influence, there was a strong influence of the processing step on TR (ANOVA; p<0.05; Tukey test) (Fig 2). Most losses occur at the grating and fermentation steps (~40% loss) and the losses are less at the subsequent steps: pressing and roasting (~15% additional loss). The global trend was that of a stepwise degradation as in Experiment A. Similarly to Experiment A, there were overall no significant differences in TR between fermented and pressed mash and this indicates that physical losses of carotenoids may not be significant during pressing.

#### Exploring factors causing carotenoid degradation

The datasets from experiments A and B were combined in order to investigate the factors influencing TR.

**Fig 3:**
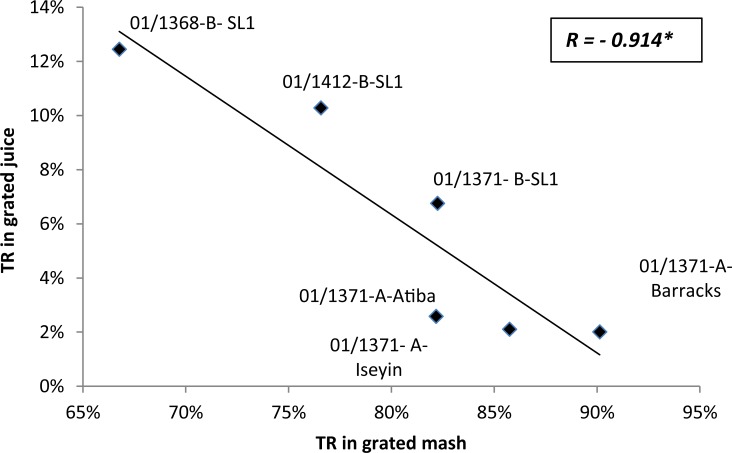
Relationships^a^ between true retention of trans-β-carotene (TR) in liquid from grated mash and in grated mash. ^a^Average of triplicate processed samples. Correlations were significant at p<0. 05 (Pearson test, two-tailed). Values for three samples in SL2 are missing.

There was a significant linear correlation (R = - 0.914) between TR in liquid from grated mash (and grated mash (Fig 3).

**Fig 4:**
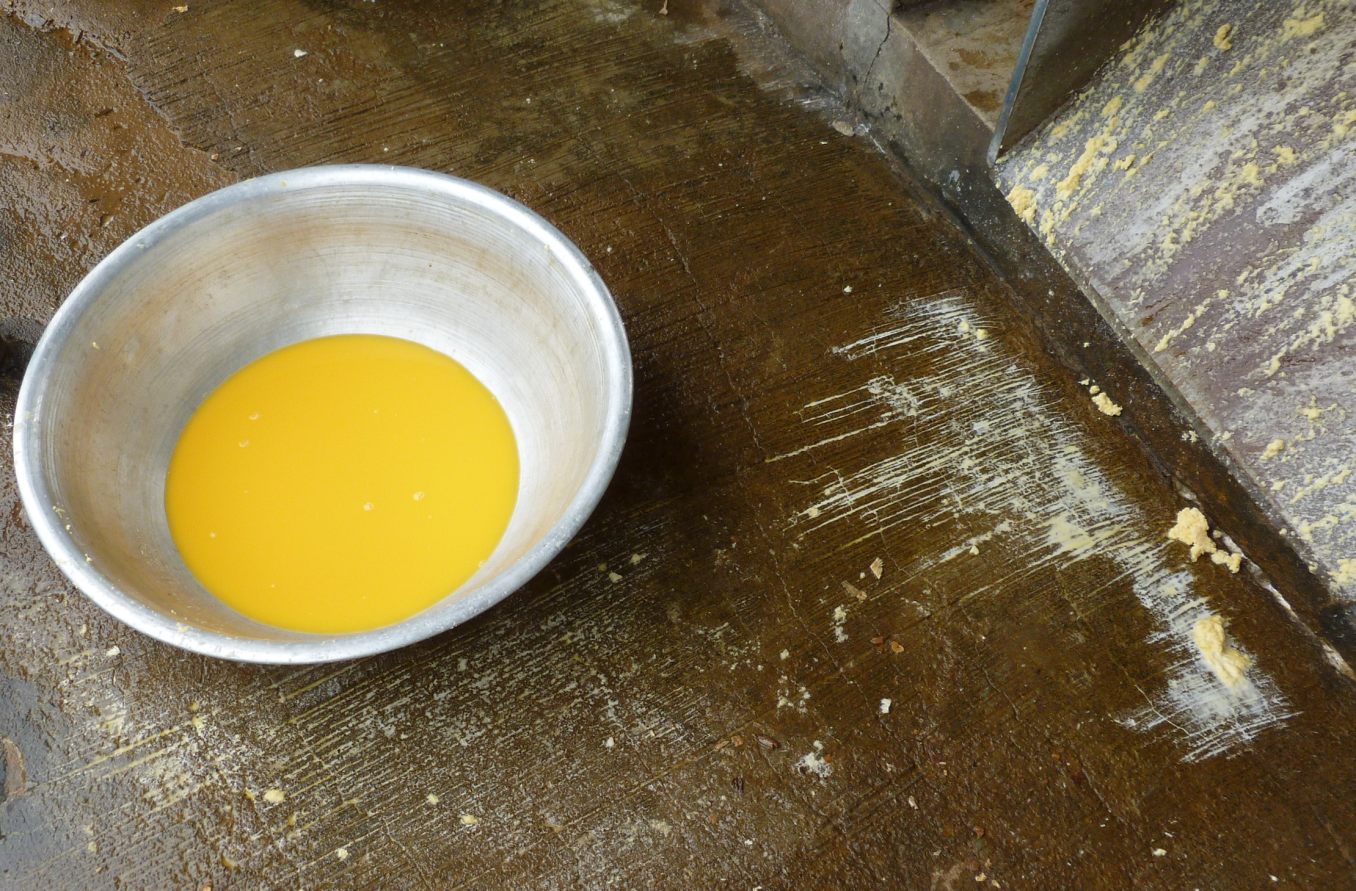
“Liquid from grated mash” freshly collected at the grating step. Source: Bechoff, A. 2012.

TR in liquid from grated mash was variable (between 2 and 13%) and the values indicate a significant loss in carotenoids in the liquid. The greater the loss of *trans*-β-carotene in mash the greater the retention in the liquid from grated mash. Because the grating step is of a short duration (2–5 minutes) (Table 1), environmental factors such as temperature and light were unlikely to cause a major loss in such a short time. Therefore it can be assumed that losses at the grating stage must result from physical losses. Visual observation of the yellow coloured liquid from the grating step also indicated a visible presence of carotenoids in the water (Fig 4). (The grey bowl on the left side of the picture contains the ‘liquid from grated mash’ of orange colour whilst a remains of the ‘grated mash’ of pale yellow colour can be observed on and around the grating equipment).

**Fig 5:**
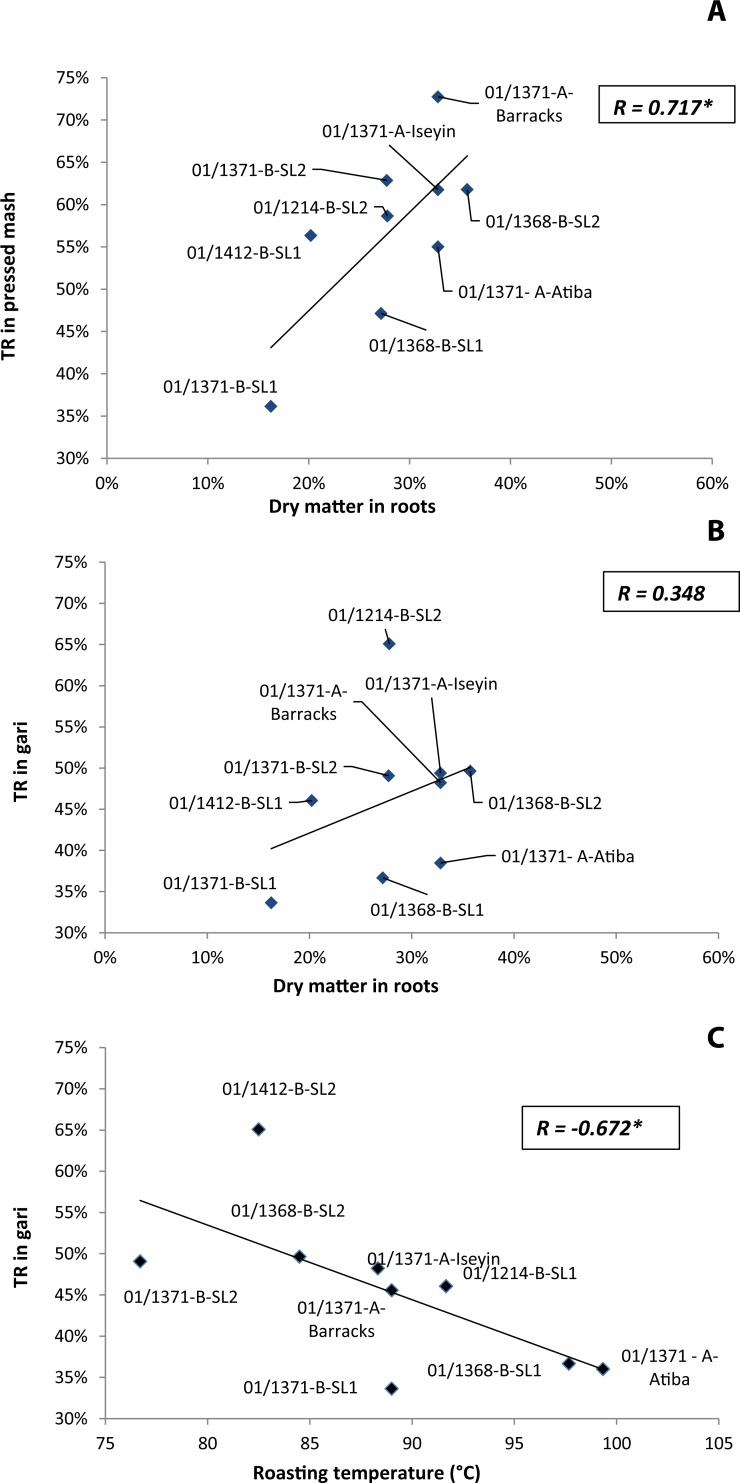
**Relationships**^**a**^ **between true retention of trans-β-carotene (TR) in pressed mash (A) and dry matter in roots; TR in gari and dry matter in roots (B); and TR in gari and roasting temperature (C).** ^a^Average of triplicate processed samples. *Correlations were significant at p<0. 05 (Pearson test, two-tailed).

Influence of different factors on TR at different steps of gari processing are presented in Fig 5.

The higher the root dry matter, the higher the TR in pressed mash (R = 0.717) (Fig 5A). On the other hand but there was no significant correlation between dry matter and TR in gari (R = 0.348) (Fig 5B).

The importance of chemical factors such as roasting temperature on TR (R = - 0.672) in illustrated in Fig 5C: the higher the roasting temperature, the lower the TR in gari: on average for a 1°C increase in temperature, there was a 1% additional *trans*-β-carotene loss.

Significant correlation between dry matter content and TR in pressed mash must result of the gari product yield (PY) that was higher in varieties with high dry matter content. However the weaker correlation between dry matter content and TR in gari shows that chemical factors during roasting could have influenced this relationship. It is suggested that roasting temperature has a significant impact on the degradation of carotenoids and this external factor could explain in part why the correlation between TR in gari and dry matter in roots was not significant. This study illustrates the complexity of separating the influence of physical and chemical factors that would conjointly influence TR at some steps of gari processing (*i*.*e*. roasting).

### Carotenoid content during gari processing

In addition to the determination of true retention (TR), the determination of provitamin A carotenoid (pVAC) content in the product is critical since pVACs relate to the nutritional value of the product that will be eventually consumed by people who are in risk of suffering of VAD.

*Trans-*β-carotene content was determined on a fresh weight basis at the different stages of processing.

#### Experiment A

Overall there was no influence of the commercial processor (Experiment A) on the *trans*-β-carotene content of the product (One-way ANOVA; p = 0.059) (Table 2).

**Table 2:** Main provitamin A carotenoid (pVAC) content (μg.g^-1^ on a fresh weight basis) at different steps of processing into gari for Experiments A^a^ & B^b^.

Experiment	A					B (SL1)				B (SL2)			
pVAC—TMS 01/1371	Trans β-carotene	13-cis-β-carotene	9-cis-β-carotene	Cis/trans	pVAC–Three varieties	Trans β-carotene	13-cis-β-carotene	9-cis-β-carotene	Cis/trans	Trans β-carotene	13-cis-β-carotene	9-cis-β-carotene	Cis/trans
**Atiba**					**TMS 01/1368**								
Peeled roots	6.21±0.39	0.09±0.01	1.33±0.04	22.9±0.8%	Peeled roots	3.83±0.12	0.97±0.05	1.83±0.05	73.1±0.5%	4.97±0.45	1.51±0.13	1.95±0.25	69.6±5.4%
Grated mash	5.24±0.06	0.09±0.01	1.38±0.06	28.1±1.3%	Grated mash	2.89±0.31	0.75±0.08	1.34±0.16	72.5±2.5%	4.34±0.16	0.63±0.64	1.60±0.09	51.2±12.5%
Fermented mash	5.32±0.66	0.09±0.01	1.38±0.15	27.2±1.9%	Fermented mash	2.69±0.52	0.64±0.12	1.02±0.25	61.7±1.6%	4.70±0.10	0.90±0.52	1.66±0.00	54.6±10.7%
Pressed & fermented mash	6.28±0.59	0.11±0.01	1.57±0.18	26.6±0.4%	Pressed & fermented mash	5.50±1.21	1.54±0.28	1.00±1.35	49.6±21.8%	5.92±0.38	1.54±0.07	1.96±0.12	59.1±1.0%
Gari	8.05±1.88	0.34±0.07	2.85±0.52	40.2±3.5%	Gari	9.10±0.92	2.87±0.22	4.14±0.40	77.1±1.2%	9.97±1.03	3.37±0.67	4.00±0.39	74.3±6.9%
**Barracks**					**TMS 01/1371**								
Peeled roots	6.21±0.39	0.09±0.01	1.33±0.04	22.9±0.8%	Peeled roots	4.21±0.09	0.99±0.04	1.69±0.06	63.6±1.6%	6.81±0.71	1.18±0.16	1.58±0.49	41.0±11.2%
Grated mash	5.97±0.05	0.10±0.02	1.50±0.08	26.9±1.5%	Grated mash	3.89±0.09	0.90±0.04	1.79±0.09	69.0±1.8%	4.35±1.79	0.95±0.72	1.35±0.54	50.0±10.9%
Fermented mash	#N/A	#N/A	#N/A	#N/A	Fermented mash	3.02±0.69	0.61±0.14	1.10±0.40	55.9±4.8%	6.47±0.51	0.83±0.70	1.68±0.07	38.8±10.6%
Pressed & fermented mash	8.69±0.32	0.10±0.01	1.80±0.12	21.9±1.2%	Pressed & fermented mash	6.14±1.35	1.39±0.32	2.38±0.59	61.2±2.1%	9.08±0.99	3.96±0.14	2.25±0.21	68.8±6.3%
Gari	10.89±0.39	0.35±0.05	3.10±0.16	31.7±1.2%	Gari	12.85±2.96	4.68±1.00	6.14±0.86	85.0±4.7%	14.52±1.93			65.2±1.6%
**Iseyin**					**TMS 01/1412**								
Peeled roots	6.21±0.39	0.09±0.01	1.33±0.04	22.9±0.8%	Peeled roots	3.57±0.09	0.89±0.02	1.65±0.06	71.3±1.3%	3.58±0.32	0.83±0.48	2.13±0.32	82.4±8.9%
Grated mash	5.53±0.35	0.11±0.01	1.59±0.05	30.9±2.1%	Grated mash	3.19±0.19	0.78±0.04	1.73±0.12	78.6±0.1%	3.53±0.33	0.81±0.41	1.72±0.12	71.6±13.9%
Fermented mash	6.03±0.20	0.11±0.01	1.70±0.09	30.1±1.2%	Fermented mash	3.66±0.41	0.89±0.13	1.87±0.41	74.9±7.7%	3.70±0.68	1.00±0.16	1.46±0.32	67.6±14.5%
Pressed & fermented mash	7.05±0.58	0.12±0.00	1.85±0.15	28.0±0.4%	Pressed & fermented mash	7.64±0.31	2.16±0.06	4.00±0.35	80.6±4.5%	5.30±0.39	1.42±0.08	2.05±0.11	65.7±7.2%
Gari	10.67±0.49	0.39±0.02	3.69±0.07	38.3±1.2%	Gari	12.88±2.81	4.65±1.05	6.48±0.90	87.0±5.3%	11.64±1.07	3.76±0.86	5.03±0.47	75.8±10.5%

Data are average ± standard deviation. Each process was conducted in triplicate

^a^ Triplicate 50kg of roots of one variety of yellow cassava TMS 01/1371 were processed into gari at three commercial gari processors (Atiba, Barracks and Iseyin) (Experiment A) and

^b^ Triplicate 25kg of roots of three different varieties of yellow cassava (01/1368; 01/1371; 01/1412) grown in two different seasons/locations (S1 and S2) were processed into gari at the IITA research station (Experiment B). Fermented mash was not collected at the Barracks.

#### Experiment B

Initial concentrations significantly varied in the roots from the three different varieties (Experiment B) (Table 2) (p<0.05). While *trans*-β-carotene is the predominant pVAC in cassava in its raw state, detectable levels of 13-*cis* and 9-*cis* isomers of β-carotene were also found in accordance with previous studies [22, 32]. Initial pVAC concentrations (on average over SL1 and SL2) were from the highest to the lowest: TMS 01/1371 (*trans*: 5.51 μg.g^-1^) with the lowest dry matter content (22% on average) > TMS 01/1368 (*trans*: 4.40 μg.g^-1^ with a dry matter of 31.4% on average > TMS 01/1412 (*trans*: 3.57 μg.g^-1^ with 24.0% of dry matter on average). In accordance with our results, Akinwale et al. [33] reported that there appear to be a genetic link between dry matter and carotenoid content in cassava roots: the varieties with the lower dry matter (or higher moisture) content had the highest initial carotenoid content. However recent data on hundreds of cassava genotypes [34] showed that there was no correlation between dry matter content and carotenoid content and therefore it is possible to identify genotypes with high carotenoid content as well as high dry matter [34]. Maroya et al. [24] working with a number of cassava clones developed in Nigeria (including the ones presented in our study) demonstrated that both natural environment (*e*.*g*. soil, climate, rainfall) and genes had an influence on total carotenoid level and also on dry matter. Moreover the interaction of environment x genes also had a significant influence on total carotenoid content in the roots and dry matter in the plant and genes may influence the stability of carotenoid-protein complexes in chromoplasts [35] and hence the TR.

During gari processing, the *trans*-β-carotene content increased (roughly two-fold) (around 10μg.g^-1^) and this was mostly because moisture was removed from the product as a result of pressing and roasting (Table 2). Increase in carotenoid content due to concentration of carotenoids in gari is in accordance with other authors’ description [20, 21, 36].

These results show that even though significant levels of pVACs were lost during gari processing, pVACs were concentrated in the final product as a result of moisture loss and this resulted in improved nutritional value of the product (gari) in terms of provitamin A content compared to the roots. In practice this means that a child who consumes 100g of biofortified gari daily would have his vitamin A daily nutritional requirements met (the calculation was based on trans-β-carotene content only. The bioconversion factor of trans-B-carotene into retinol is 5:1 [11] and the Estimated Average Requirement (EAR) for a child under five years of age is200 μg retinol equivalent [37]). Gari can be consumed as it is (snack) or made into dough by adding boiling water (eba). In the later process, further carotenoid losses in the dough may occur but those may be minimal if boiling water is simply added to gari and the product stirred into a dough.

### *Cis*-isomers and *cis*-isomerisation during gari processing

Under stressful conditions such as heating and UV-light exposure, *trans*-carotenoids tend to isomerise into *cis*-carotenoids. *Cis*-isomerisation may be considered as a negative effect of processing since *cis*-isomers have a lower provitamin A activity (about half) than that of *trans*-β-carotene [13].

#### Experiment A

Processor type (Experiment A) also had a significant influence on the *cis/trans* ratio (Table 2) with Barrack centre having significantly fewer *cis*-isomers formed than Atiba and Iseyin centres (25.8%; 29.0% and 30.0% respectively): slightly less *cis*-isomerisation may be explained by shorter processing time and therefore less exposure to temperature and light at Barrack.

There was a significant effect of the step of processing on the *cis*-isomerisation (ANOVA; p<0.05). Percent of *cis*-isomers (both 13-*cis* and 9-*cis*) over *trans*-isomers significantly increased due to roasting for the commercial processors: (before roasting: 25.5%; after roasting: 36.7%, on average). This was in accordance with previous work on boiling and frying of cassava [38, 39] that also showed an increase in *cis*-isomers (9-*cis* and 13-*cis*). Thakkar et al. [22] observed that gari processing was associated with a decline in all-*trans*-β-carotene and concomitant increase in 13-*cis*-β-carotene as observed in our study. Marx et al. [40] working on effect of thermal processing on *cis*-isomerisation in carrot containing preparations further demonstrated that that the higher the roasting temperature the greater the percent of *cis*-isomers; this was not clearly shown in our study and this might be because other factors such as roasting time would have to be accounted for.

#### Experiment B

Additionally there was a significant varietal effect (ANOVA; p<0.05) on *cis*-isomerisation (Experiment B): variety TMS 01/1412 proportionally had significantly more *cis*-isomers than 01/1368 that had significantly more *cis*-isomers than 01/1371 (ANOVA; Tukey test; p<0.001) (*cis/trans* ratio was 75.5%; 64.8% and 59.9%, respectively) (Table 2). Varietal influence is interesting because it shows that not only the process is responsible for *cis*-isomerisation but naturally present *cis*-isomers in cassava can be found in different proportions as this was reported by Carvalho et al. [35].

Furthermore there was an interaction between variety and processing steps on *cis*-isomerisation (p<0.05). Interaction of variety and processing will make it difficult to predict how *trans* and *cis*-isomers carotenoids in cassava varieties will vary during gari processing [38].

## Conclusions

We found that True Retention in *trans*–β-carotene (TR) under unset conditions is similar to other studies under set conditions found in literature (TR ~ 50%) and that therefore losses are confirmed to be high during gari processing from biofortified cassava under field conditions. Those significant losses of pVACs were explained to be the result of a combination of physical losses of pVACs and chemical losses (oxidation). Physical losses are demonstrated to be mainly resulting of carotenoid leaching in the water *i*.*e*. at the grating step: because of the grating conditions (short time, ambient temperature), it is unlikely that chemical factors could be responsible for such significant losses at this stage. The carotenoid loss pattern suggests that initially TR decreases quickly for a small amount of water removed from the product (during grating and also fermenting), then in further steps TR decreases more slowly for more water removed (during pressing) and finally at the roasting step TR decreases because of chemical oxidation due to high temperatures during roasting.

These findings imply that physical carotenoid loss from the extracting liquids should be reduced in order to optimise TR. Gari is by nature a dry product and retaining more moisture in the final product therefore cannot be proposed as a solution. One option may be to collecting and drying soluble solids containing carotenoid from the water lost. Another alternative may be to increase the dry matter content of the roots since this decreases the amount of moisture contained in the roots and therefore the moisture squeezed during the process. As a result the product yield (PY) of gari could be improved and higher PY of gari means higher TR since it is calculated based on the weight of the product, and also a higher gari PY will be beneficial for businesses who buy roots and process them into commercial gari. This work shows that physical losses in carotenoids should be accounted for in studies on retention.

## Supporting information

S1 Data Raw data used for this publication. (XLSX)
